# Supplementary material for: mTORC2 confers neuroprotection and potentiates immunity during virus infection
Source: Nat Commun. 2021 Oct 14;12:6020. doi: 10.1038/s41467-021-26260-5 (PMC8516965; doi:10.1038/s41467-021-26260-5)
Supplement: Supplementary file 1 — Supplementary Information [file 41467_2021_26260_MOESM1_ESM.pdf]

Supplementary Information

mTORC2 confers neuroprotection and potentiates immunity during virus infection

Rahul K. Suryawanshi,[1] Chandrashekhar D. Patil, [1] Alex Agelidis,[1,2] Raghuram Koganti,[1] Joshua M. Ames,[1,2] Lulia Koujah,[1,2] Tejabhiram Yadavalli,[1] Krishnaraju Madavaraju,[1] Lisa M. Shantz,[3] Deepak Shukla[1,2]*

Affiliations
1 Department of Ophthalmology and Visual Sciences, University of Illinois at Chicago, Chicago IL, USA
2 Department of Microbiology and Immunology, University of Illinois at Chicago, Chicago, IL 60612, USA
3 Department of Cellular and Molecular Physiology, Pennsylvania State University College of Medicine, Hershey, PA, USA.
* Correspondence: dshukla@uic.edu

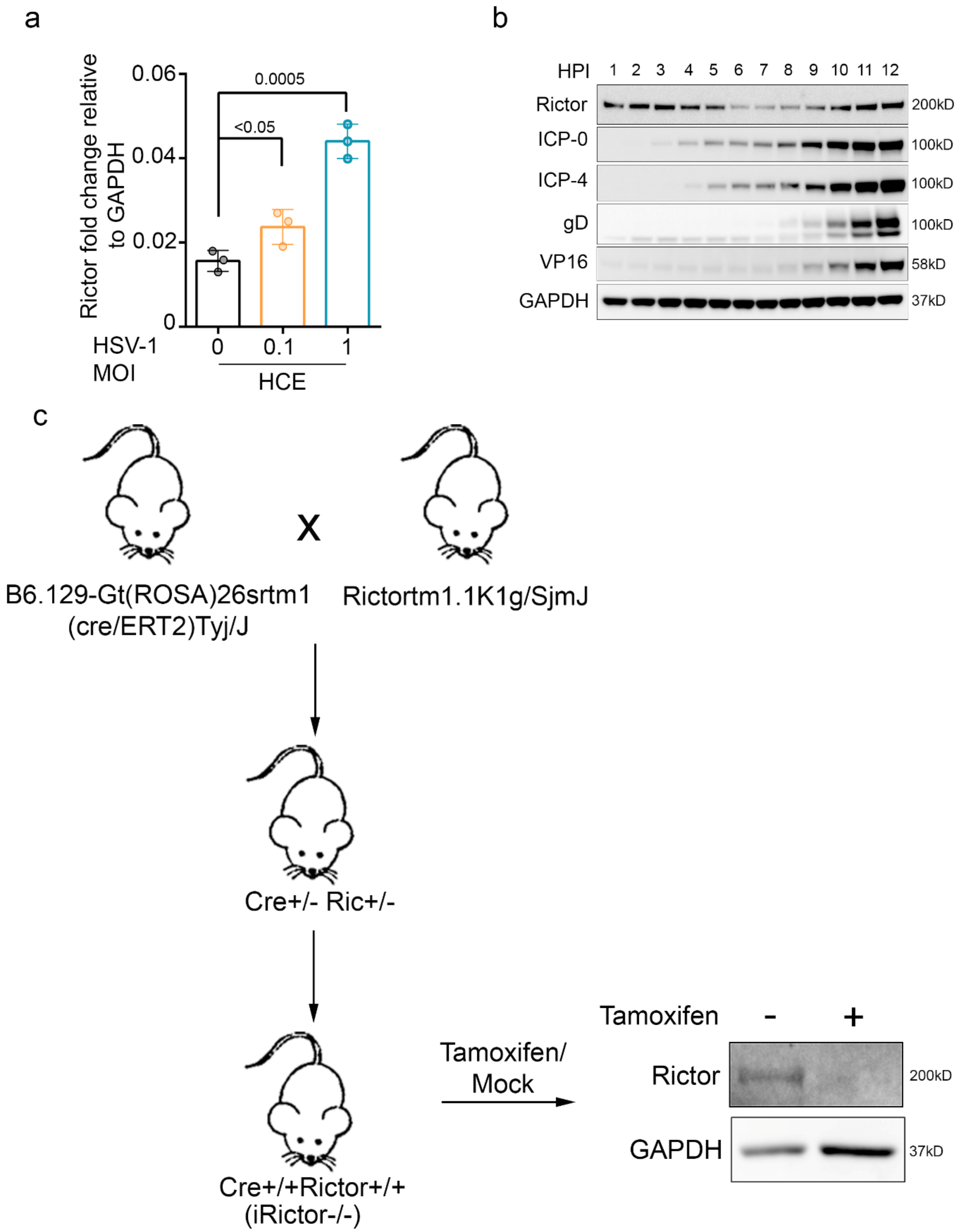

Supplementary Fig. 1 Rictor expression during HSV-1 infection. a, qRTPCR analysis showing increase expression of Rictor with respect to HSV-1 infection in Human corneal epithelial cells (n=3). Two-tailed unpaired t-test was used to analyze the data. Data represented as mean ± SEM. No adjustments were made for multiple comparisons. **b,** A representative image of protein

expression in HSV-1 (1 multiplicity of infection) infected HCE cells at different time points. **c,** Schematic for the development of conditional rictor knockout mouse model. Data in the micrograph b-c is representative of three independent experiments. Source data underlying Fig. 1b and 1c are provided as a Source Data file.

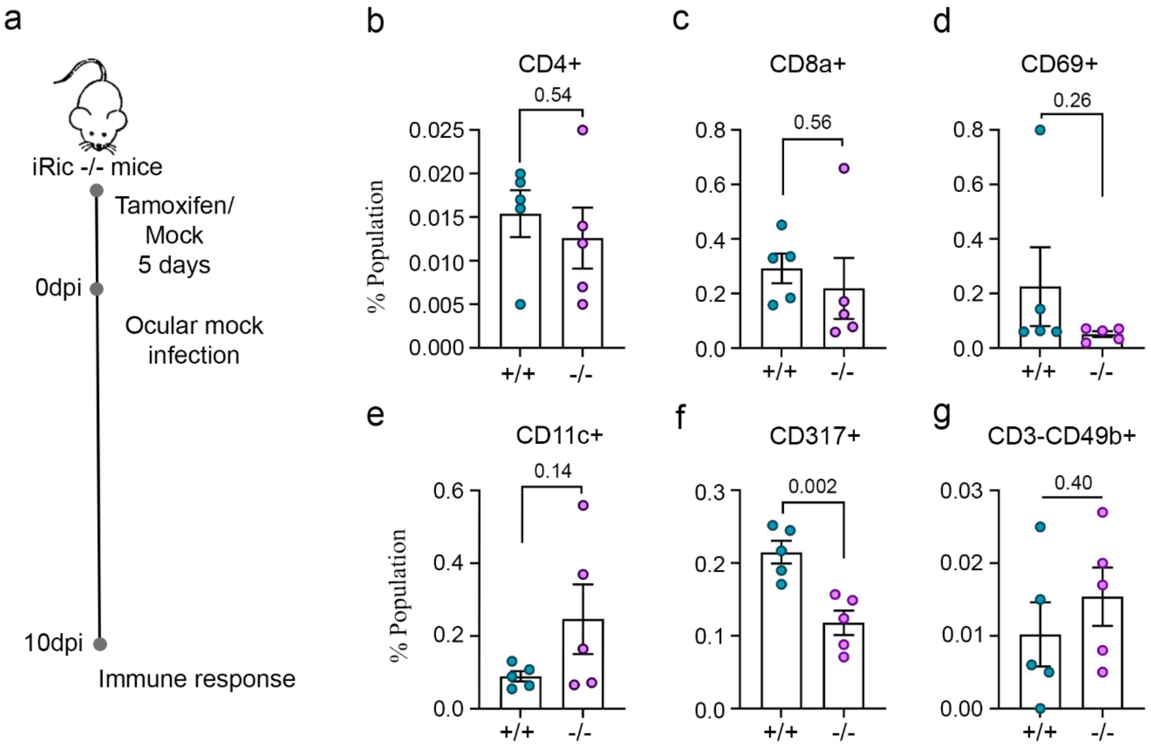

Supplementary Fig. 2 Immune response in mock infected iRic +/+ and iRic-/- animals. a. Schematics of the experiment showing conditional knockout of the iRic-/- mice using Tamoxifen or mock. The mice were mock infected and immune response on the ocular tissue was evaluated. **b-g,** Graph representing population of respective immune cells in mock infected eye at 12dpi (n=5). Two-tailed unpaired t-test was used to analyze the data presented in b-g. Data are represented as mean ± SEM in b-g.

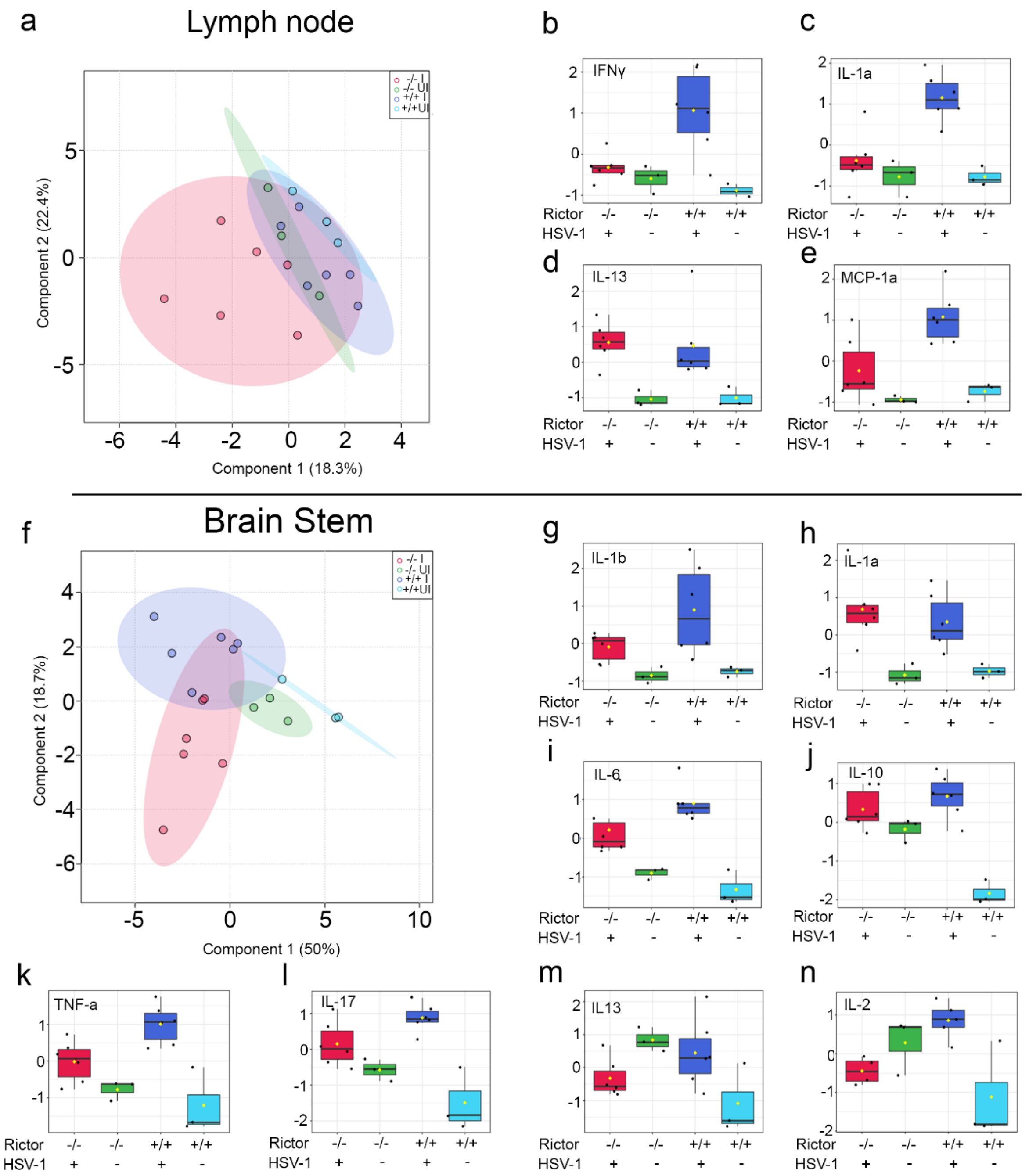

Supplementary Fig. 3 Cytokine levels in lymph node and brain stem tissue show differences in iRic +/+ or -/- mice. a, Principle component analysis of cytokine expression in lymph node tissue of HSV-1 infected mice **b-e,** A graph showing expression of respective cytokines in lymph node of HSV-1 or mock infected iRic +/+ or -/- mice. **f.** Principle component analysis of cytokine expression in brain stem tissue of HSV-1 infected mice **g-n.** A graph showing expression of respective cytokines in brain stem of HSV-1 or mock infected iRic +/+ or -/- mice. The bar plots in b to e and g to n show the normalized values (mean +/- one standard deviation). The boxes range from the 25% and the 75% percentiles; the 5% and 95% percentiles are indicated as error bars;

single data points are indicated by circles. Medians are indicated by horizontal lines within each box.

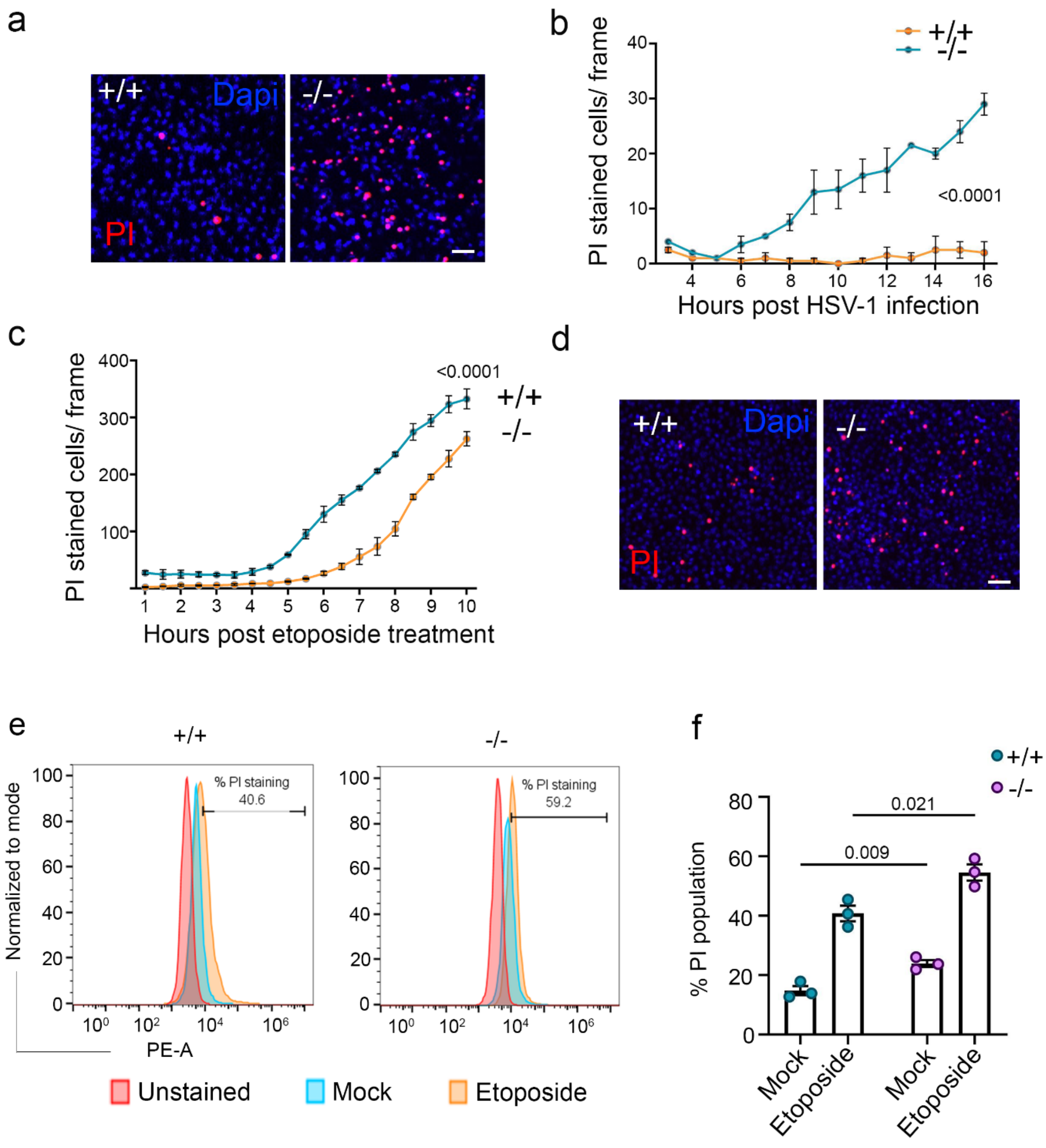

Supplementary Fig. 4 iRic-/- cells are sensitive to cell death with HSV-1 infection or etoposide treatment. a. Representative micrograph of fluorescent imaging for HSV-1 infected iRic +/+ or -/- MEFs stained with propidium iodide (red) and DAPI (blue) at 12hpi. Scale bar- 50µm **b.** Graph showing number of cells stained with PI at indicated time points (n=3). Data represented as mean ± SEM in b-c and the dataset between the two groups have been compared using 2way ANOVA.

c. Graph showing number of iRic +/+ or -/- MEF cells treated with etoposide and stained with PI at indicated time points (n=3) **d.** Representative micrograph of time point fluorescent imaging of iRic +/+ or -/- MEFs treated with etoposide at 8h post treatment. Scale bar- 50µm e. Representative micrograph showing percent PI stained population in mock or etoposide treated iRic +/+ or -/- MEFs at 8h post treatment. f. quantification of flow cytometry data represented in e. (n=3), Two-tailed unpaired t-test was used to analyze the data presented in f. Data represented as mean ± SEM in f. Data in the micrograph a and d is the representative of three independent experiments.

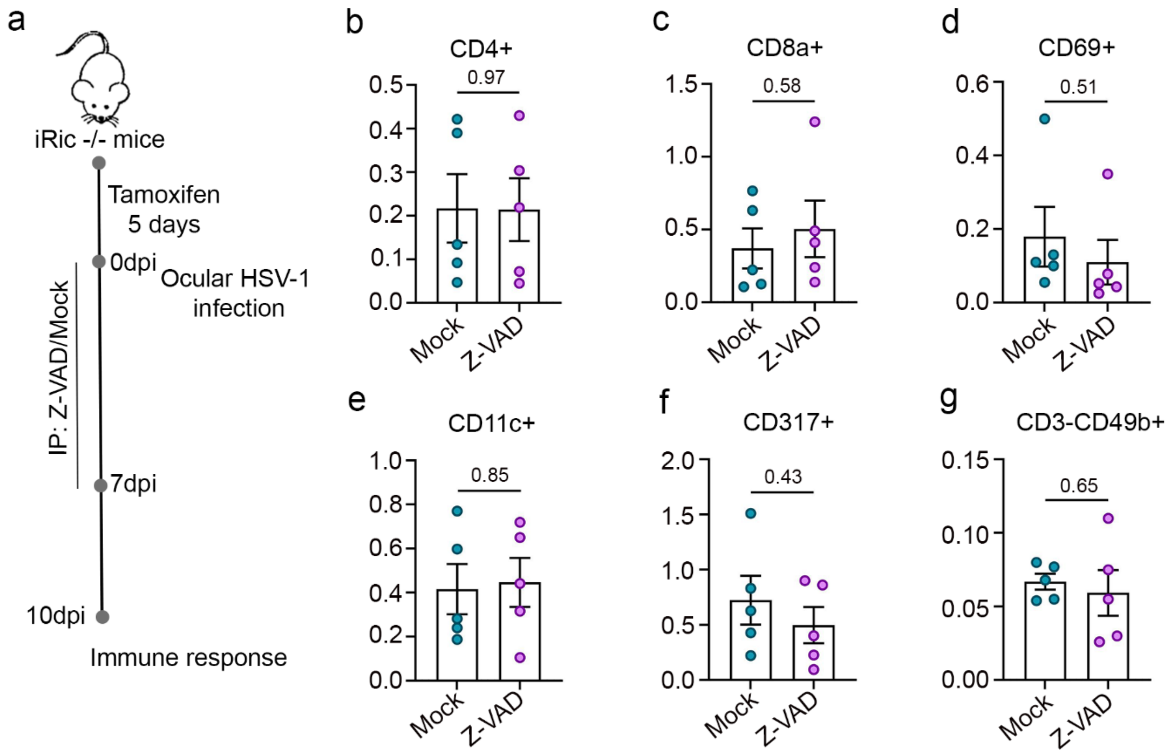

Supplementary Fig. 5 Immune response in mock infected iRic +/+ and iRic-/- animals. a, Schematics of the experiment showing conditional knockout of the iRic-/- mice using Tamoxifen. The animals were infected with HSV-1 and treated with either Z-VAD or mock. The immune response was analyzed at day 10 post infection. **b-g,** Graph representing population of respective immune cells in mock infected eye at 10dpi (n=5). (n=3), Two-tailed unpaired t-test was used to analyze the data presented in b-g. Data are represented as mean ± SEM in b-g.

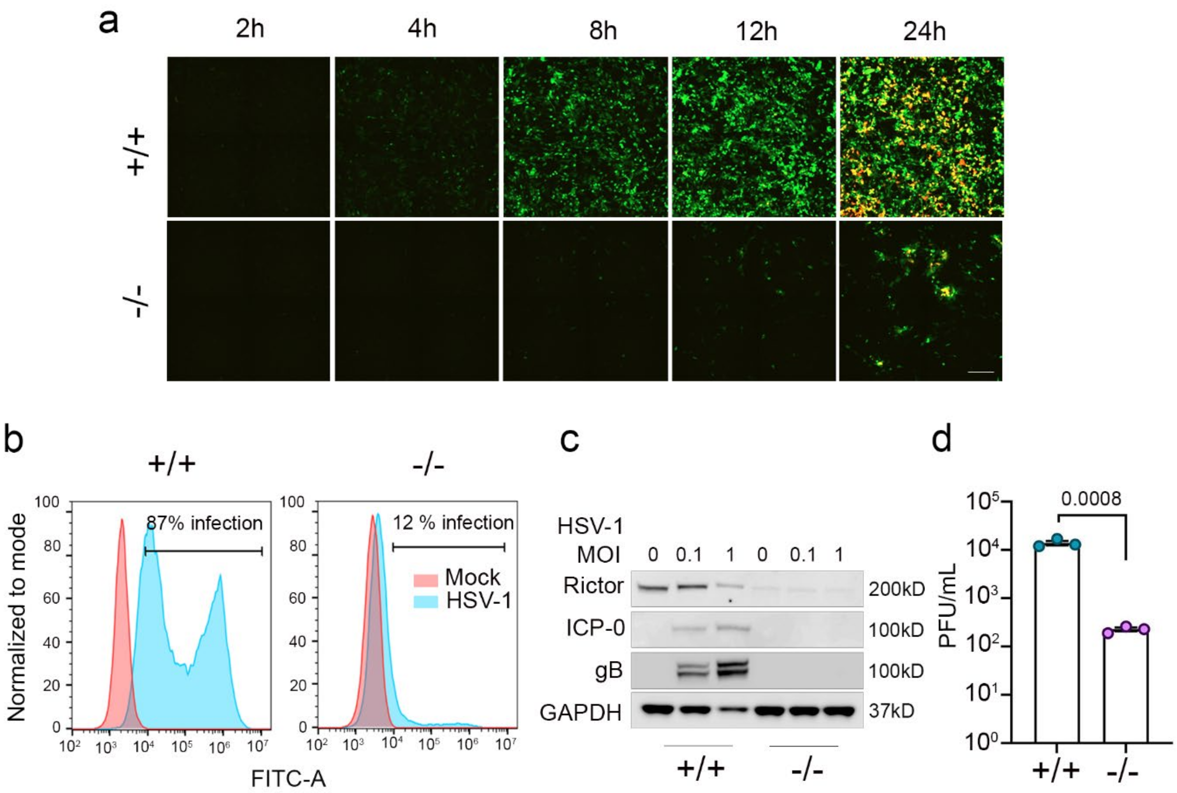

Supplementary Fig. 6 iRic-/- cells restricts virus replication. a, Representative micrograph of fluorescent imaging for HSV-1 infected iRic +/+ or -/- MEFs. Data is representative of three independent experiments. Scale bar 50μm. **b,** Representative micrograph of flow data showing percent of virus infected iRic +/+ or -/- MEFs. **c,** Representative micrograph of western blot showing HSV-1 virus protein expression for ICP-0 and gB. **d,** Viral plaque assay indicating plaque forming units per milliliter (PFU/mL) (n=3), Two-tailed unpaired t-test was used to analyze the data. Data represented as mean ± SEM in d. Source data underlying Fig. 6c are provided as a Source Data file.

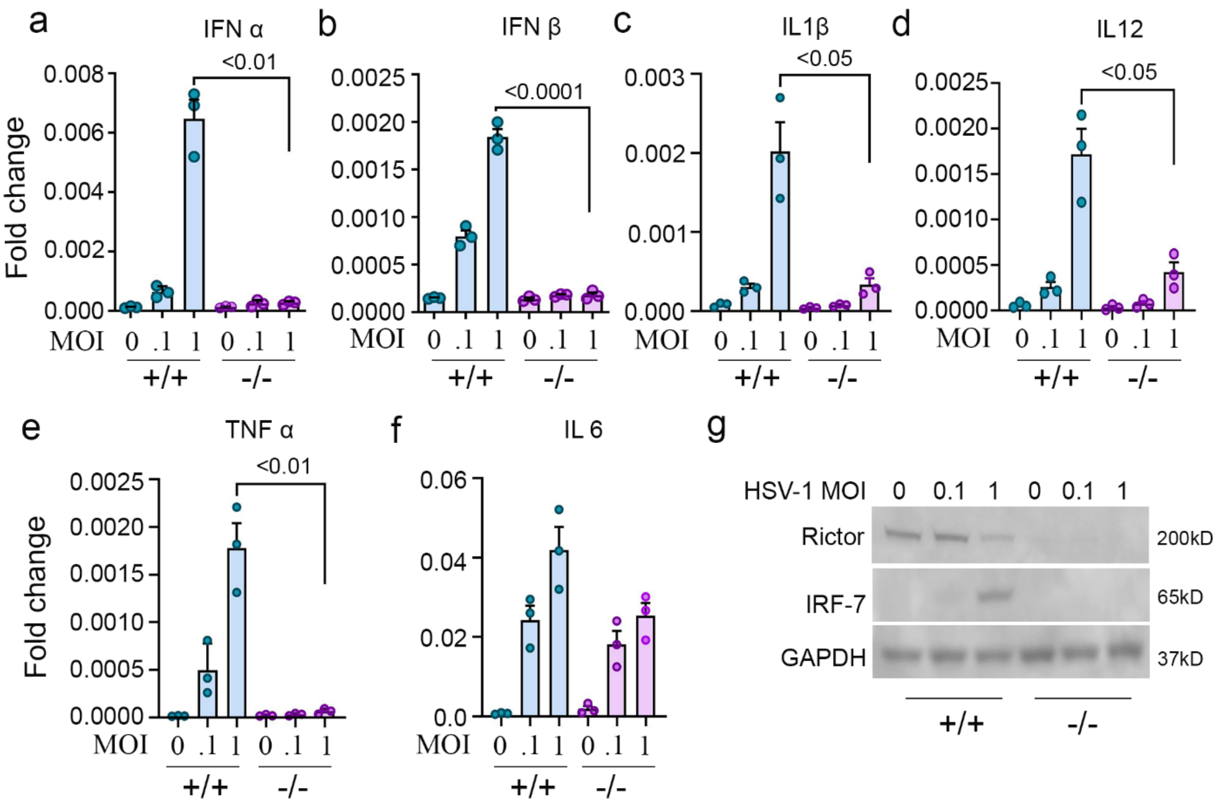

Supplementary Fig. 7 Rictor depletion reduces cytokine transcription in-vitro. Fold change of transcripts for **a,** IFN-α **b,** IFN-β **c,** IL-1β **d,** IL-12 **e,** TNF-α and **f,** IL-6 (n=3), Two-tailed unpaired t-test was used to analyze the data presented in a-f. Data are represented as mean ± SEM in a-f. **g,** Representative micrograph of western blot for IRF-7 protein expression in HSV-1 infected MEFs. Data is representative of three independent experiments. Source data underlying Fig.7g are provided as a Source Data file.

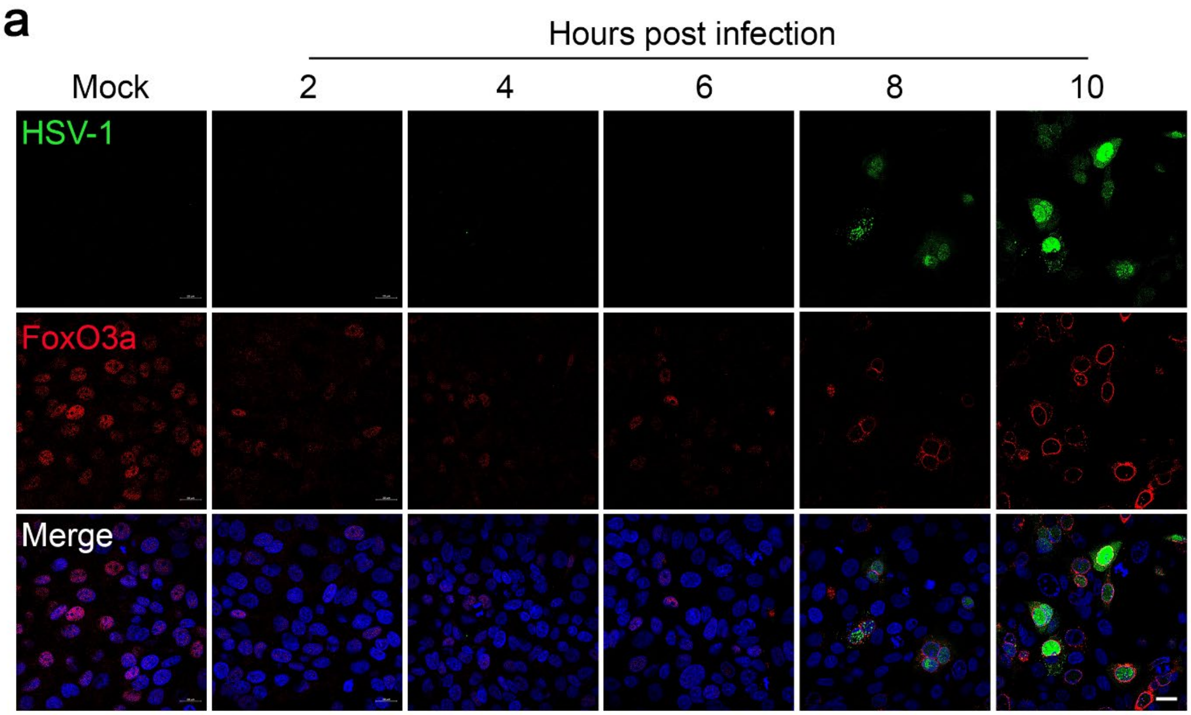

Supplementary Fig. 8 Time point analysis of FoxO3a localization in HSV-1 infected HCE. a. Representative micrograph of immunofluorescence confocal imaging illustrating expression and location of FoxO3a protein in HSV-1 infected HCE cells. Images were taken at different hpi. Data is representative of three independent experiments. Scale bar 20μm.

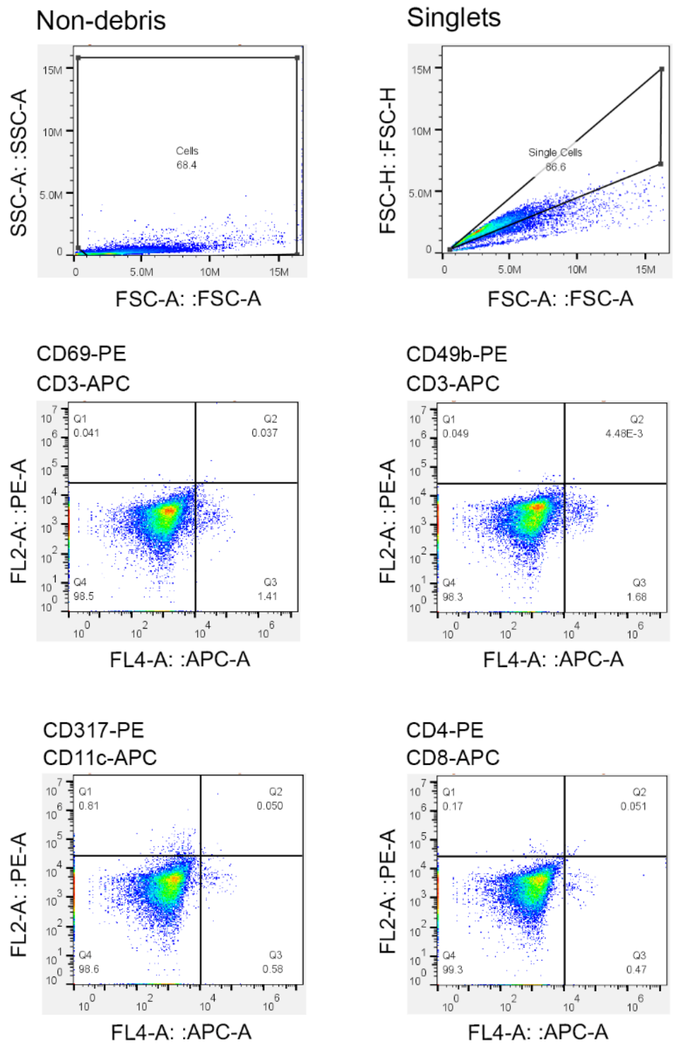

Supplementary Fig. 9 Representative flow cytometry getting strategy. In gating strategy, the dead cell population was excluded in in P1. The cells were sorted on the basis of their scatter properties with Forward (FSC) and side scatter (SSC) to give an idea of the size and granularity of the cells (P2). The P2 population was further divided into sub-populations based on surface markers as shown in the representative dot plot (P3). The boundaries between positive and negative population were determined by quadrant.

Supplementary Table 1: The list of primers.

Primer	Sequence
HSV-1 Glycoprotein D qPCR forward:	5′- TACAACCTGACCATCGCTTC-3′
HSV-1 Glycoprotein D qPCR reverse:	5′- GCCCCCAGAGACTTGTTGTA-3′
HSV-1 ICP-0 qPCR forward:	5′- ACAGACCCCCAACACCTACA-3′
HSV-1 ICP-0 qPCR reverse:	5′- GGGCGTGTCTCTGTGTATGA-3′
HSV-1 Glycoprotein B qPCR forward:	5′- GCCTTTTGTGTGTGTGTGGG-3′
HSV-1 Glycoprotein B qPCR reverse:	5′- GCCTTTTGTGTGTGTGTGGG-3′
Human GAPDH qPCR Forward:	5′- TCCACTGGCGTCTTCACC-3′
Human GAPDH qPCR reverse:	5′- GGCAGAGATGATGACCCTTTT-3′
Human Rictor qPCR Forward:	5′- TGGGTGTGAACCATGAGAAGTATG-3′
Human Rictor qPCR reverse:	5′- GGTGCAGGAGGCATTGCT-3′
Mouse interferon-α qPCR Forward:	5′-CCTGCTGGCTGTGAGGAAAT-3′
Mouse interferon-α qPCR reverse:	5′-GACAGGGCTCTCCAGACTTC-3′
Mouse interferon-β qPCR Forward:	5′- TGTCCTCAACTGCTCTCCAC-3′
Mouse interferon-β qPCR reverse:	5′- CATCCAGGCGTAGCTGTTGT-3′
Mouse interleukin-6 qPCR forward:	5′-ACGGCCTTCCCTACTTCACA-3′
Mouse interleukin-6 qPCR reverse:	5′-CATTTCCACGATTTCCCAGA-3′
Mouse interleukin-12 qPCR forward:	5′-AAATGAAGCTCTGCATCCTGC-3′
Mouse interleukin-12 qPCR reverse:	5′-TCACCCTGTTGATGGTCACG-3′
Mouse TNF-α qPCR forward:	5′-GCCTCTTCTCATTCCTGCTTG-3′
Mouse TNF-α qPCR reverse:	5′-CTGATGAGAGGGAGGCCATT-3′
Mouse β-actin qPCR forward:	5′- CGGTTCCGATGCCCTGAGGCTCTT-3′
Mouse β-actin qPCR reverse:	5′- CGTCACACTTCATGATGGAATTGA-3′
