# Supplementary material for: mTORC2 confers neuroprotection and potentiates immunity during virus infection
Source: Nat Commun. 2021 Oct 14;12:6020. doi: 10.1038/s41467-021-26260-5 (PMC8516965; doi:10.1038/s41467-021-26260-5)
Supplement: Supplementary file 3 — Reporting summary [file 41467_2021_26260_MOESM3_ESM.pdf]

Corresponding author(s): Dr. Deepak Shukla
Last updated by author(s): 2021/07/09

Reporting Summary

Nature Portfolio wishes to improve the reproducibility of the work that we publish. This form provides structure for consistency and transparency in reporting. For further information on Nature Portfolio policies, see our Editorial Policies and the Editorial Policy Checklist.

Statistics

For all statistical analyses, confirm that the following items are present in the figure legend, table legend, main text, or Methods section.

n/a	Confirmed	
☐	☒	The exact sample size (n) for each experimental group/condition, given as a discrete number and unit of measurement
☐	☒	A statement on whether measurements were taken from distinct samples or whether the same sample was measured repeatedly
☐	☒	The statistical test(s) used AND whether they are one- or two-sided<br>*Only common tests should be described solely by name; describe more complex techniques in the Methods section.*
☐	☒	A description of all covariates tested
☐	☒	A description of any assumptions or corrections, such as tests of normality and adjustment for multiple comparisons
☐	☒	A full description of the statistical parameters including central tendency (e.g. means) or other basic estimates (e.g. regression coefficient) AND variation (e.g. standard deviation) or associated estimates of uncertainty (e.g. confidence intervals)
☐	☒	For null hypothesis testing, the test statistic (e.g. F, t, r) with confidence intervals, effect sizes, degrees of freedom and P value noted<br>*Give P values as exact values whenever suitable.*
☒	☐	For Bayesian analysis, information on the choice of priors and Markov chain Monte Carlo settings
☒	☐	For hierarchical and complex designs, identification of the appropriate level for tests and full reporting of outcomes
☒	☐	Estimates of effect sizes (e.g. Cohen's d, Pearson's r), indicating how they were calculated

Our web collection on statistics for biologists contains articles on many of the points above.

Software and code

Policy information about availability of computer code

Data collection	BD Accuri C6 Plus vl.0, ZEN Black, and Biotek GENS v3.04 Imager softwares were used for data collection in this study.
Data analysis	Graph Pad Prism 8, FlowJo vl0.0.7 were used for data analysis in this study.

For manuscripts utilizing custom algorithms or software that are central to the research but not yet described in published literature, software must be made available to editors and reviewers. We strongly encourage code deposition in a community repository (e.g. GitHub). See the Nature Portfolio guidelines for submitting code & software for further information.

Data

Policy information about availability of data

All manuscripts must include a data availability statement. This statement should provide the following information, where applicable:

- Accession codes, unique identifiers, or web links for publicly available datasets
- A description of any restrictions on data availability
- For clinical datasets or third party data, please ensure that the statement adheres to our policy

Data and materials used in this study can be made available through contact with the corresponding author.

Field-specific reporting

Life sciences study design

All studies must disclose on these points even when the disclosure is negative.

Sample size	All in vitro experiments have been carried out in triplicates and for in vivo experiments the n value has been provided in figure legend section. There is only one report available for role of mTORC2 on HSV-1 infection (Sato et al. 2018. https://doi.org/10.1038/s41590-018-0203-2) therefore the data is limited to perform a power analysis to estimate the number of mice needed in virus infection group, for this reason the sample size for in vivo experiments was determined based on previous report (Sato et al. 2018. https://doi.org/10.1038/s41590-018-0203-2).
Data exclusions	No data were excluded from study
Replication	All the experiments have been carried out in triplicates to confirm the successful reproducibility of the data.
Randomization	For animal experiment the 6-8 week old animals were used. The animals were allocated randomly to the mock or HSV-1 infection group. The in vitro model and mice were grouped by genotypes and compared between two genotypes and HSV-1 infection.
Blinding	The researcher performing the animal experiment was blind for the animal groups. The groups were code labeled by lab manager which were reveled after results analysis. For in vitro cell culture studies blinding was not relevant to this study as there was minimal scoring, and most measures were quantifiable by standard cellular or biochemical assays.

Reporting for specific materials, systems and methods

We require information from authors about some types of materials, experimental systems and methods used in many studies. Here, indicate whether each material, system or method listed is relevant to your study. If you are not sure if a list item applies to your research, read the appropriate section before selecting a response.

Materials & experimental systems

n/a	Involved in the study
☐	☒ Antibodies
☐	☒ Eukaryotic cell lines
☒	☐ Palaeontology and archaeology
☐	☒ Animals and other organisms
☒	☐ Human research participants
☒	☐ Clinical data
☒	☐ Dual use research of concern

Methods

n/a	Involved in the study
☒	☐ ChIP-seq
☐	☒ Flow cytometry
☒	☐ MRI-based neuroimaging

Antibodies

Antibodies used	For Western blot: HSV-1 viral proteins were detected by Mouse monoclonal [5H7] anti-HSV1 ICP0 (ab6513), gB (ab6506) both purchased from Abcam (Cambridge, United Kingdom. Following antibodies were used for western blot or immunofluorescence were FoxO3a (2497S), AKT (9272S), p-FoxO3aS253 (9466S), Rictor (2114S), p-FoxO1T24/p-FoxO3AT32 (9464S), p-AKTS473 (9271S), FoxO1 (FoxO1), Histone (4499S), IRF7 1:500 (4920) all purchased from cell signaling technology (Danvers, MA), GAPDH (10494-1-AP) was purchased from Proteintech Group, Inc., (Rosemont, IL). Mouse monoclonal [7B] anti-GAPDH (Santa Cruz), Goat anti-Mouse IgG (H+L) Highly Cross-Adsorbed Secondary Antibody, Goat anti-Rabbit IgG (H+L) Cross-Adsorbed Secondary Antibody. For flow cytometry: Flow antibodies were purchased from Biolegend were CD3 (100236), CD69 (104507), CD11b (101206), CD11c (117310), CD49b (108907), CD317 (127104) and those purchased from Tonbo biosciences, San Diego were CD4 (50-0042-U100) and CD8a (20-18886-U100).
Validation	All primary antibodies were confirmed or the species and application through the validation statement on the manufacturer's website and their use in the literature. We are providing here the list of antibodies used and a respective PubMed id citation mentioning their use.<br>Anti-mouse ICP0 (ab6513) : PMID: 29263259<br>Anti-mouse gB (ab6506) : PMID: 29870688<br>Anti-Rabbit FoxO3a (2497S) : PMID: 31729980<br>Anti-Rabbit AKT (9272S) : PMID: 33187537<br>Anti-Rabbit p-FoxO3aS253 (9466S) : PMID: 30798853<br>Anti-Rabbit Rictor (2114S) : PMID: 31356902<br>Anti-Rabbit FoxO1 (FoxO1) : PMID: 31784498<br>Anti-Rabbit IRF7 (4920) : PMID: 29042669<br>Anti-Rabbit GAPDH (10494-1-AP) : PMID: 28901402ure research | reporting summary April 2020<br>Anti-mouse CD69 PE-conjugated monoclonal (104507) : PMID: 28521278<br>Anti-mouse CD11b APC-conjugated monoclonal (101206) : PMID: 28784846<br>Anti-mouse CD11c APC-conjugated monoclonal (117310) : PMID: 29079107<br>Anti-mouse CD49b PE-conjugated monoclonal (108907) : PMID: 28741259

Anti-mouse CD317 PE-conjugated monoclonal (127104) : PMID: 31089128
Anti-Rabbit p-FoxO1T24/p-FoxO3AT32 (9464S) : PMID: 31331823
Anti-Rabbit p-AKTS473 (9271S) : PMID: 33052929

Eukaryotic cell lines

Policy information about cell lines

Cell line source(s)	1. Human corneal epithelial cell line (RCB1834 HCE-T) was procured from Kozaburo Hayashi (National Eye Institute, Bethesda, MD).<br>2. iRictor conditional knock out mouse embryonic fibroblasts were isolated from mice having Rictor conditional alleles able to transiently express tamoxifen-inducible Cre recombinase (CreERT2). Rictor knockout in the cells was achieved by treating them with 4-hydroxytamoxifen (4OHT) (2 μM) for 72h.<br>3. MEFs with FoxO4+/-, p53-/- immortalized FoxO3a+/+ and FoxO3a-/- were a generous gift from Prof. Nissim Hay (University of Illinois at Chicago, USA).<br>4. LUHMES cells (ATCC) were provided by Dr. David Bloom (University of Florida)] [HCE cells were provided by Dr. Kozaburo Hayashi (National Eye Institute, Bethesda, MD).<br>5. VERO cells were a generous gift from Prof. Patricia Spear, NorthWestern University, Chicago
Authentication	iRictor conditional knock out MEF's, FoxO4+/-, p53-/- immortalized FoxO3a+/+ and FoxO3a-/- MEF's were tested for absence of expression of Rictor and FoxO3a by western blotting and after authentication they were used for experiment. The data is provided in the results section of the manuscript.<br>Cell lines were authenticated using STR analysis
Mycoplasma contamination	All the cell lines were tested negative for mycoplasma contamination.
Commonly misidentified lines (See ICLAC register)	No commonly misidentified cell lines were used in the study.

Animals and other organisms

Policy information about studies involving animals; ARRIVE guidelines recommended for reporting animal research

Laboratory animals	Laboratory animals used: RictorF/F mutant mice possessing loxP sites flanking exon 11 of the RPTOR independent companion of MTOR, complex 2 (Rictor) gene (STOCK Rictortm1.1Klg/SjmJ) and whole body cre/ERT2 mice (B6.129-Gt(ROSA)26Sortm1(cre/ERT2)Tyj/J) were purchased from The Jackson Laboratory. The mice were bred to create a inducible Rictor knockout mice (iRic-/-). In order to knock out Rictor from iRic-/- animals, they were treated with tamoxifen (2mg/kg) for five days and then used for the experiment.<br>Sex: Female,<br>Age 6-8 week old
Wild animals	Study did not involve wild animals
Field-collected samples	Study did not involve samples collected from fields
Ethics oversight	All animal care and procedures were performed in accordance with the institutional and NIH guidelines, and approved by the Animal Care Committee at University of Illinois at Chicago (ACC protocol 17-077).

Note that full information on the approval of the study protocol must also be provided in the manuscript.

Flow Cytometry

Plots

Confirm that:

- [x] The axis labels state the marker and fluorochrome used (e.g. CD4-FITC).
- [x] The axis scales are clearly visible. Include numbers along axes only for bottom left plot of group (a 'group' is an analysis of identical markers).
- [x] All plots are contour plots with outliers or pseudocolor plots.
- [x] A numerical value for number of cells or percentage (with statistics) is provided.

Methodology

Sample preparation	The animal tissue were digested with collagenase and the cells were sieved with 70µm filter. The cells were washed with flow buffer and labeled with flow antibodies for 1hr on ice. The unstained cells and cell stained with single color antibody were used for gating purpose.
Instrument	The immunolabelled cells were analyzed with Accuri C6 Plus flow cytometer (BD Biosciences).

Software	BD Accuri C6 Plus software and Treestar FlowJo v10.0.7 were used for all flow cytometry data analysis.
Cell population abundance	Cell abundance in post-sort fraction was ~50,000.
Gating strategy	A simple gating strategy was used to gate all cells based on SSC and FSC areas, then single cells based on FSC height and FSC area, then single cells were analyzed for a one or two color stain experiment.

☒ Tick this box to confirm that a figure exemplifying the gating strategy is provided in the Supplementary Information.
